# Taxonomic revision of *Mcvaughia* W.R.Anderson (Malpighiaceae): notes on vegetative and reproductive anatomy and the description of a new species

**DOI:** 10.3897/phytokeys.117.32207

**Published:** 2019-02-05

**Authors:** Rafael F. Almeida, Isabel R. Guesdon, Marcelo R. Pace, Renata M.S. Meira

**Affiliations:** 1 Universidade Federal de Minas Gerais, Programa de Pós-Graduação em Biologia Vegetal, Avenida Antonio Carlos 6627, CEP 31270-901, Belo Horizonte, MG, Brazil Universidade Federal de Minas Gerais Belo Horizonte Brazil; 2 Universidade Federal de Viçosa, Programa de Pós-Graduação em Botânica, Departamento de Biologia Vegetal, CEP 36570-900, Viçosa, Minas Gerais, Brazil Universidade Federal de Viçosa Viçosa Brazil; 3 Universidade Federal do Amazonas, Instituto de Ciências Exatas e Tecnologia, 69103-128, Itacoatiara, Amazonas, Brazil Universidade Federal do Amazonas Itacoatiara Brazil; 4 Universidad Nacional Autónoma de México, Instituto de Biología, Departamento de Botánica, Circuito Exterior, Ciudad Universitaria, Coyoacán, 04510, Mexico City, Mexico Universidad Nacional Autónoma de México Mexico City Mexico; 5 Smithsonian Institution, National Museum of Natural History, Department of Botany, 10th Street & Constitution Avenue NW, 20560, Washington DC, USA National Museum of Natural History, Smithsonian Institution Washington United States of America

**Keywords:** Atlantic Forest, Brazil, Caatinga, Malpighiales, Neotropical flora

## Abstract

A taxonomic revision of *Mcvaughia* is presented, including the description of a new species from the state of Piauí, Brazil, and notes on wood, secondary phloem, leaf, and floral morpho-anatomy. We present a key to the species, full morphological descriptions, a distribution map, and notes on distribution, ecology, etymology, and conservation status for each species.

## Introduction

*Mcvaughia* W.R.Anderson is a genus of Malpighiaceae comprising three species endemic to the Atlantic Forest and Caatinga domains in northeastern Brazil (Anderson 1979; Amorim and Almeida 2015), one of which is described here for the first time. The genus can be easily recognized by its shrubby habit, anterior petals nestled inside one another, horseshoe-shaped anthers, and drupes with the epicarp twisted in a 180° angle (Anderson 1979; Amorim and Almeida 2015; this study). The genus was first described almost 40 years ago as monospecific, based on collections from the northern state of Bahia, Brazil (Anderson 1979). At that time, *Mcvaughia* was placed in the subfamily Byrsonimoideae by Anderson (1979), due to its habit, subulate styles with minute stigmas, tricolpate pollen grains, and chromosome numbers of 6 to 12 (Anderson 1977). Within this subfamily, the genus was morphologically related to *Burdachia* A.Juss. and *Glandonia* Griseb., sharing characteristics such as the posterior petal bearing glandular margins (Anderson 1981).

The first phylogenetic studies for Malpighiaceae demonstrated that a clade comprising the genera *Burdachia*, *Glandonia*, and *Mcvaughia* made the Byrsonimoideae paraphyletic (Cameron et al. 2001; Davis et al. 2001; Davis and Anderson 2010). Since then, those genera have been placed in the so-called Mcvaughioid clade, one of the three early diverging lineages of Malpighiaceae: Byrsonimoids, Acridocarpoids, and Mcvaughioids (Davis and Anderson 2010). The Mcvaughioid clade comprises eight species distributed in three genera: *Burdachia*, *Glandonia*, and *Mcvaughia* (Anderson 1981; Reis e Silva 2007; Amorim and Almeida 2015; Guesdon et al. 2018). About three decades later the description of *Mcvaughia*, the second species of the genus was discovered and described for the restinga vegetation and coastal dunes from the northern state of Sergipe, Brazil (Amorim and Almeida 2015).

During recent visits to Brazilian herbaria, we found a third species of *Mcvaughia* endemic to seasonally dry forests from the state of Piauí, Brazil. We present a taxonomic revision of *Mcvaughia*, including full morphological descriptions, a distribution map, illustrations, and notes on conservation, distribution, and etymology of all species accepted in the genus. Additionally, we present a detailed anatomical description of wood, bark, leaves, and flowers for the genus. This is the first of a series of joint studies focusing on the biosystematics of Malpighiaceae by the Malpighiales Biosystematics Working Group (GEBIM 2018).

## Methods

### Taxonomy

Morphological and phenological data were based on herbaria samples (ALCB, ASE, CEN, CEPEC, F, FLOR, G, HST, HUEFS, K, MBM, MICH, NY, P, RB, SP, TEPB, U, UB, US, and VIC; herbaria acronyms according to Thiers, continuously updated). The indumentum terminology follows Anderson (1981), structure shapes follow Radford et al. (1974), the inflorescence terminology and morphology follows Weberling (1965, 1989), and fruit terminology follows Spjut (1994) and Anderson (1981). Wood and bark anatomical descriptions follow the recommendations of the IAWA Committee for hardwoods and barks (IAWA Committee 1989, Angyalossy et al. 2016). The conservation status was proposed following the recommendations of IUCN Red List Categories and Criteria, Version 3.1 (IUCN 2012). GeoCAT (Bachman et al. 2011) was used for calculating the Extent of Occurrence (EOO) and the Area of Occurrence (AOO). Maps were elaborated using ArcGIS 9.3 software (ESRI 2010), and geographical coordinates were obtained from herbaria specimens and the literature (Anderson 1979; Amorim and Almeida 2015).

### Anatomy

Fresh samples of leaves, inflorescence, and stems were fixed in the field with FAA (formaldehyde, acetic acid and 50% ethyl alcohol; 1:1:18, by volume) for 48h (Johansen 1940) and vouchers deposited at HUEFS, and VIC herbaria (acronyms according to Thiers 2018, continuously updated). Additional herborized specimens were sampled for leaf and floral anatomy: *Mcvaughiabahiana* [D. Cardoso 57 (CEPEC), M. L. Guedes 12148 (CEPEC), I. Silva-Guesdon 300, 301(VIC)], *M.sergipana* [I. Silva-Guesdon 305, 306 (VIC)], and *M.piauhiensis* [R. Barros 2922 (HUEFS)]. Herbarium samples were rehydrated according to Smith and Smith (1942), dehydrated in an ethanol series and stored in 70% ethanol. Leaves and petals were also submitted to clearing following Vasco, Thadeo, Conover, Daly (2014, modified), to dissociation techniques (Franklin 1945), and mounted in glycerin jelly (Johansen 1940). Samples stored in ethanol were then embedded in methacrylate resin (Historesin Leica; Leica Microsystems, Heidelberg, Germany) and sectioned using an automatic rotary microtome (Leica RM2265, or USA Leica RM2155, Deerfield, USA). Longitudinal and cross-sections were stained with toluidine blue at pH 4.7 (O’Brien and McCully 1981) and mounted in resin (Permount, Fisher Scientific, NJ, USA).

For wood and bark anatomy, *Mcvaughiasergipana* stems [specimen Amorim 8393 (HUEFS)] were boiled in water and glycerin for a month to soften its extremely stiff wood (Angyalossy et al. 2016), being subsequently embedded in polyethylene glycol 1500 (PEG 1500; Rupp 1964), and sectioned in a sliding microtome with a permanent hard steel knife type “C” (Barbosa et al. 2018) with the aid of a Styrofoam resin (Barbosa et al. 2010). The sections were double stained in Safrablau (Bukatsch 1972 modified by Kraus and Arduin 1997) and mounted in Canada balsam. Imaging was performed using a light microscope (AX70TRF; Olympus Optical, Tokyo, Japan) equipped with a digital camera (AxioCam HRc; Zeiss, Göttingen, Germany). Anatomical analyses of leaves and flowers were conducted at the Plant Anatomy Laboratory – UFV and wood and bark anatomy at the Plant Anatomy Laboratory of the Smithsonian Natural History Museum (SI-NMNH).

### SEM analysis

Micromorphological data were obtained using a scanning electron microscope (SEM) at the Center for Microscopy and Microanalysis, Universidade Federal de Viçosa. Fixed samples were dehydrated in an ethanol series, submitted to the critical point technique (CPD 020; Bal-Tec, Balzers, Liechtenstein), sputter coated with gold (Bozzola and Russell 1992), and observed and photographed using a Leo 1430VP SEM (Zeiss, Cambridge, United Kingdom). The anatomical patterns of secretory structure were described as sessile when the secretory epidermis covered all the projected area; subsessile when the secretory epidermis is surrounded by non-secretory epidermis; and stalked when a short stalk is present.

## Results

### Mcvaughia

Taxon classificationPlantaeMalpighialesMalpighiaceae

W.R.Anderson, Taxon 28: 157. 1979

#### Type species.

*Mcvaughiabahiana* W.R.Anderson

#### Description.

*Shrubs* to subshrubs, perennial, growing in sandy soils. *Branches* cylindrical, densely sericeous or lanate-velutinous, generally glabrescent at age. *Stipules* epipetiolar, completely connate, persistent. *Leaves* opposite; petiole eglandular; blade bearing 2–many glands abaxially. *Thyrsi* terminal, pedunculate, many-branched; cincinni alternate to subopposite, 1–14-flowered; bracts persistent; bracteoles persistent, one of them 1-glandular, the other eglandular, gland green in bud turning yellow in anthesis. *Flowers* zygomorphic; floral buds slightly flattened at middle; pedicel stout, straight in bud. *Sepals* leaving petals exposed in pre-anthesis, all 2-glandular. *Petals* bright to golden yellow, glabrous, the anterior two remaining cupped one inside the other; lateral petals with the margin erose; posterior petal bearing several marginal glands. *Stamens* 7–8, staminodes 2–3 (stamens opposite the posterior-lateral sepals and the posterior petal); filaments glabrous, those opposite the posterior-lateral petals slightly curved towards the apex; connectives inconspicuous; anthers horseshoe-shaped, glabrous, outer locules confluent at apex, reduced to antherodes in staminodes. *Ovary* 3-carpellate, 3-locular, 2 locules ± anterior, apparently collapsed lacking ovules, 1 locule almost posterior, fertile, 1-ovulate; styles 3, slender, truncate to uncinate at apex; stigma minute, lateral. *Drupes* rugose, twisted, asymmetric, 1-locular, proximal chamber thick-walled, 1-seeded, distal chamber thin-walled, filled with a viscous secretion (allowing the fruit to float and to be dispersed by water).

#### Etymology.

The epithet pays tribute to Dr. Rogers McVaugh (*1909–2009†), an American botanist, expert in the Mexican flora.

#### Distribution.

*Mcvaughia* is restricted to sandy soils within sedimentary basins of Northeastern Brazil, with different species being endemic to each sedimentary basin: *Mcvaughiabahiana* – Tucano basin, *M.piauhiensis* – Parnaiba basin, and *M.sergipana* – Sergipe-Alagoas basin (Amorim and Almeida 2015). Sedimentary basins represent conspicuous phytogeographic zones within the Caatinga domain, with a distinct biota from other areas of Caatinga over crystalline shield, holding endemism records for some angiosperm families (Cardoso and Queiroz 2007; Almeida et al. 2018; Silva and Souza 2018).

#### Biogeography.

*Mcvaughia* is the sister-group of *Burdachia* and *Glandonia*, comprising a lineage (*Mcvaughia* clade) of early diverging Malpighiaceae with water-dispersed fruits, most commonly found growing along wetland floodplains and upland forest throughout the Amazon Basin (Anderson 1981). Davis et al. (2014) estimated that the ancestor of this clade might have arisen around 38.0-33.9 Myr, and its extant lineages diversified from 25 to 15 Myr. The description of *M.piauhiensis* corroborates the hypothesis of Amorim and Almeida (2015) that extant lineages of *Mcvaughia* have probably experienced recent radiations along the caatingas and restingas of Northeastern Brazil. This inference is based on the fact that all three *Mcvaughia* species occur along current or past courses of the São Francisco river (SFR): *M.piauhiensis* occurs to the west, and *M.bahiana* and *M.sergipana* occur to the east near the SFR’s delta (Fig. 11). During the upper Pliocene, the SFR flowed to the equatorial Atlantic Ocean via the Piauí or Canindé Rivers, in the Parnaiba river basin, state of Piauí (King 1956; Nascimento et al. 2013). However, the rising of the Grande and Ibiapaba Ridges in northwestern Ceará state during the Pliocene/Pleistocene boundary would have abruptly interrupted the SFR’s course (King 1956; Mabesoone 1994; Nascimento et al. 2013; Almeida et al. 2018). By this time, the ancestor of *Mcvaughia* might have already been established in this area, and its populations might have been isolated from each other by the formation of several lakes in northwestern Bahia, due to the interruption of the river flow (King 1956; Mabesoone 1994; Nascimento et al. 2013). It was not until the Mindel glaciation (ca. 450.000 years ago) that the SFR found its way to the eastern Atlantic Ocean, bordering the states of Alagoas and Sergipe (King 1956; Mabesoone 1994; Nascimento et al. 2013), thus, paving the way for recent vicariant cladogenesis events within these populations.

#### Wood Anatomy of *Mcvaughiasergipana*.

**Figure 1.**
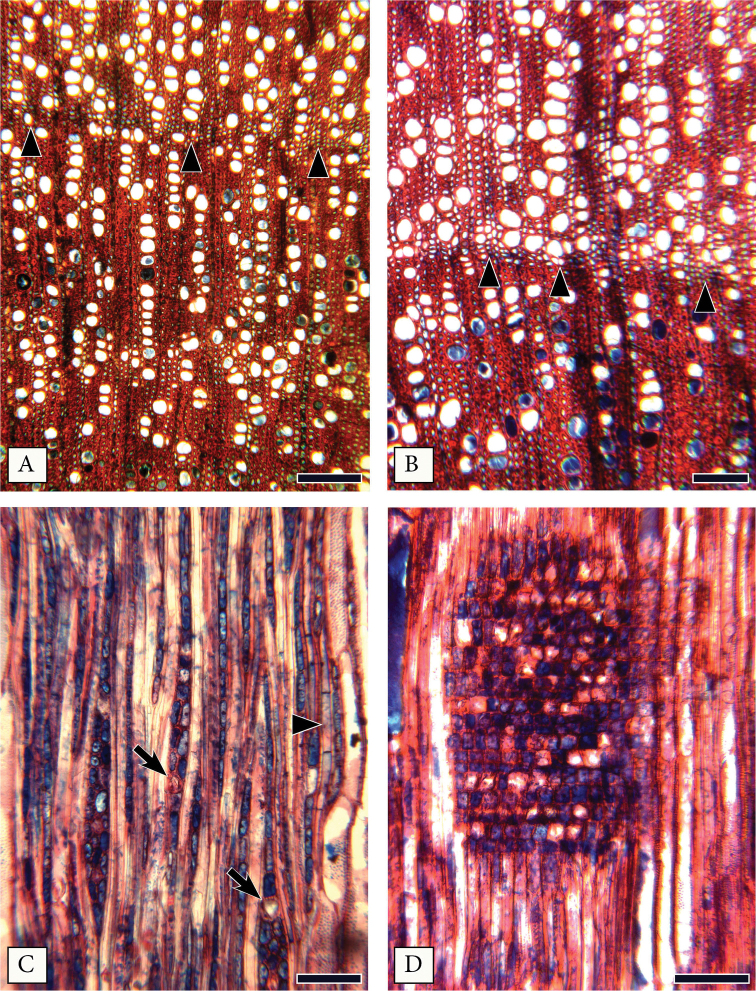
Wood anatomy of *Mcvaughiasergipana*. **A–B** transverse sections: Growth rings marked by radially narrow fibers (arrowheads) and a discontinuous line of axial parenchyma (in B); vessels are narrow and abundant, arranged in radial rows of 4 or more cells; some solitary vessels present; parenchyma rare, paratracheal scanty or at the growth ring limits; heartwood vessels in the bottom with content **C** radial section: Rays 2–3 cells wide, non-storied; prismatic crystals present in ray cells (arrows); parenchyma with 3 cells per strand (arrowhead) **D** ray heterocellular with procumbent, square and upright cells mixed throughout the ray. Scale bars: 150 μm (**A**), 100 μm (**B–C**), 60 μm (**D**).

Heartwood and sapwood indistinct light brown; grain straight to slightly wavy. In anatomical sections, the heartwood appears with abundant cell contents (bottom of Fig. 1A–B) in vessels and other cell types. The contents stain in blue with safrablau (Fig. 1A–B). Growth ring boundaries are distinct (Fig. 1A–B), marked by a line of axial parenchyma (Fig. 1B) and thicker walled, radially narrow fibers (Fig. 1A–B). Wood is diffuse-porous (Fig. 1A). Vessels are narrow, 34 ± 9 μm, generally arranged in radial multiples of 4 or more (Fig. 1A), abundant, 244 ± 57 vessels/mm^2^, and with a mean length of 365 ± 73 μm. Some solitary vessels and shorter radial multiples are also present (Fig. 1A–B). Perforation plates are simple. Intervessel pits are alternate, minute, 4 μm, vestured. Vessel ray-pitting with distinct borders; similar to intervessel pits in size and shape. Fibers very thick-walled (Fig. 1A–B), with simple to minutely bordered pits. Axial parenchyma paratracheal, scanty and forming a discontinuous line at the growth ring limits (Fig. 1A–B), 3–4 cells per parenchyma strand (Fig. 1C). Rays with 1–3 cells wide (Fig. 1C), lower than 1 mm (Fig. 1C), heterocellular mixed, with procumbent, square and upright cells mixed throughout the ray (Fig. 1C). Perforated ray cells common, non-storied (Fig. 1C). Large prismatic crystals in enlarged ray cells (Fig. 1C) of the body and margins.

#### Secondary phloem anatomy of *Mcvaughiasergipana*.

**Figure 2.**
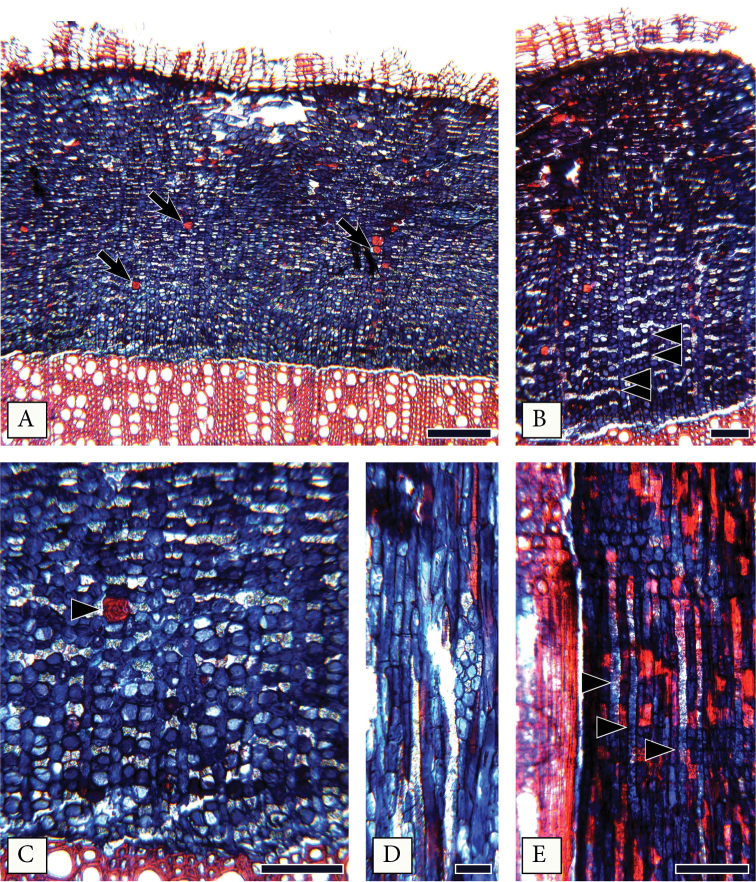
Secondary phloem of *Mcvaughiasergipana*. **A–C** Transverse section: **A** Phloem non-stratified, with scattered fiber-sclereids (arrows); Rays dilating slightly **B** Crystalliferous axial parenchyma arranged in diffuse-in-aggregate narrow bands (arrowhead) **C** Crystalliferous axial parenchyma with druse crystals, forming diffuse in aggregate bands, isolated fiber-sclereids present (arrowhead) **D** A ray 3 cells wide, fiber-sclereids and axial parenchyma in tangential section **E** Bands of crystalliferous parenchyma with druses evident also in radial section; Rays heterocellular mixed. Scale bars: 200 μm (**A**), 100 μm (**B–C, E**), 50 μm (**D**).

Growth rings are indistinct, phloem non-stratified (Fig. 2A–C). Conducting phloem represents a narrow band of 11–12 cells away from the cambium. Nonconducting phloem is marked by the collapse of the sieve tubes, a more significant dilatation of the axial and ray parenchyma (Fig. 2A–B), and belated sclerosis of some scattered ray cells. Sieve tubes are diffuse (Fig. 2C), solitary or in radial multiples of 2 cells (Fig. 2C), sieve plates are simple, slightly inclined, 441 ± 71 μm^2^ in area (24 ± 2 μm in diameter). Sieve tube elements length is 243 ± 45 μm. Slime plug always evident at the sieve plates. One to two companion cells per sieve tube element, as seen in transverse section. When two companion cells are present, one lies on each side of the sieve tube. Dilatation due to cell expansion and division is evident in the rays, axial parenchyma, and the cortex (Fig. 2A–B). Axial phloem parenchyma constitutes the ground tissue (Fig. 2A–C), four cells per parenchyma strand. Axial parenchyma with druse crystals appears as diffuse-in-aggregate lines (Fig. 2A–C, E), giving a stratified appearance to the phloem, clear both in transverse and radial section (Fig. 2A–C, E). Rays with a straight course, slightly dilated (funnel-shaped). Ray width, height, and composition equal to those of secondary xylem (Fig. 2D–E). Sclerenchyma is represented by scattered bulky fiber-sclereids (Fig. 2A–C) and a few sclerified ray cells in the nonconducting phloem (Fig. 2B). Phloem elements are non-storied. Druses are abundant, present in absolutely all parenchyma cells (Fig. 2C). Crystals are present both in chambered axial parenchyma cells (Fig. 2C) and in individual axial and ray parenchyma cells of the phloem (Fig. 2D) and cortex.

#### Leaf anatomy.

**Figure 3.**
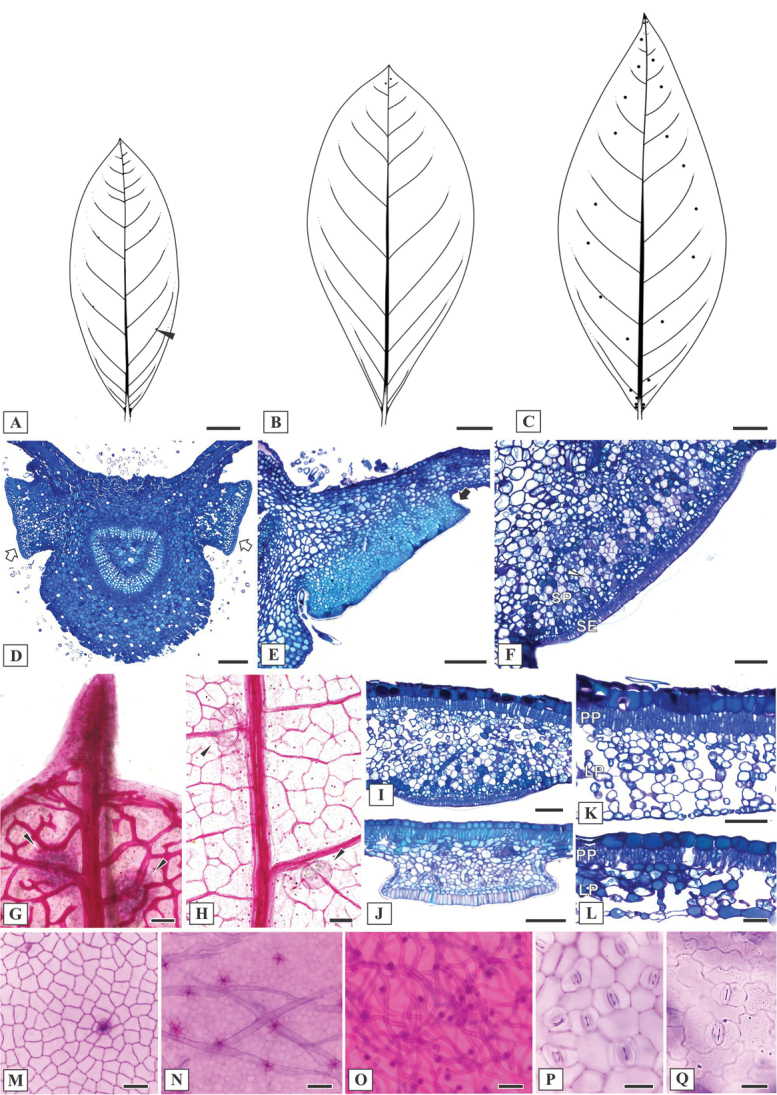
Leaf morphoanatomy of *Mcvaughia* species. **A** patterns of leaf glands distribution on the abaxial leaf surface of *M.bahiana***B** patterns of leaf glands distribution on the abaxial leaf surface of *M.piauhiensis***C** patterns of leaf glands distribution on the abaxial leaf surface of *M.sergipana***D** transverse section of leaf base showing the basilaminar pair of stalked glands (white arrows) **E** basilaminar leaf gland with a stalk (black arrow) in *M.piauhiensis***F** basilaminar gland in *M.sergipana* showing a sessile position (SE= anatomical arrangement with secretory epidermis, SP= vascularized secretory parenchyma) **G–H** laminar glands on the apex of cleared leaves of *M.sergipana* and *M.bahiana* respectively, note the apical tooth (**G**) **I** sessile laminar glands in *M.sergipana***J** stalked laminar gland in *M.piauhiensis***K–L** transverse sections of the leaf blade; mesophyll with uniserial palisade-like parenchyma and spongy parenchyma composed by several or few layers in *M.sergipana* and *M.bahiana*, respectively; note the idioblast with druse crystals at the mesophyll (white arrow) and the stomata distribution at the abaxial leaf surface (black arrow) **M–N** adaxial epidermis surface of *M.piauhiensis* and *M.sergipana*, showing scars of malpighiaceous trichomes **O** abaxial epidermis surface of trichomes abundance in *M.bahiana***P–Q** outline of the anticlinal epidermal cell walls: straight in *M.sergipana* (**P**) and sinuous in *M.bahiana* (**Q**). Laminar scale bars: 1 cm (**A–C**), 100 μm (**D, F–K, N–O**), 150 μm (**E**), 50 μm (**L–M, P–Q**).

The leaf anatomy in the species of *Mcvaughia* is quite similar. The vascular system of petioles is arranged as a curved arc with two conspicuous accessory bundles. The most distinctive leaf character is the distribution pattern of glands (Fig. 3A–C). These glands are distributed at the base and throughout the leaf blade, varying in number from two to eight basilaminar glands and from few to several distal laminar glands. The basilaminar and laminar leaf glands were identified as short-stalked (Fig. 3D–E), except in *M.sergipana* which were recognized as sessile and partially set in the mesophyll (Fig. 3F). Based on the anatomical arrangement, these leaf glands are composed of a palisade-like secretory epidermis and vascularized parenchyma (Fig. 3F). The laminar glands in *M.sergipana* are conspicuous (Fig. 3G, I), while in *M.bahiana* (Fig. 3H) and *M.piauhiensis* (Fig. 3J) these glands are inconspicuous, and difficult to see with the naked eye. The leaf blade anatomy revealed a dorsiventral mesophyll with a single layer of palisade parenchyma and a variable number of spongy parenchyma layers. In *M.sergipana* the spongy parenchyma has several layers (Fig. 3K), while in *M.bahiana* and *M.piauhiensis* fewer layers were observed (Fig. 3L). Idioblasts containing druses are commonly observed in the mesophyll (Fig. 3L). Malpighiaceous trichomes were observed in the epidermis of both surfaces (Fig. 3M–O), being more abundant abaxially and in young leaves. The outline of the anticlinal epidermal cell walls adaxially is straight (Fig. 3M–N), while abaxially may vary from straight to sinuous (Fig. 3P–Q), being exclusively straight in *M.sergipana*. All species of *Mcvaughia* show hypostomatic leaves (Fig. 3K–L), with paracytic stomata (Fig. 3P–Q).

#### Floral anatomy.

**Figure 4.**
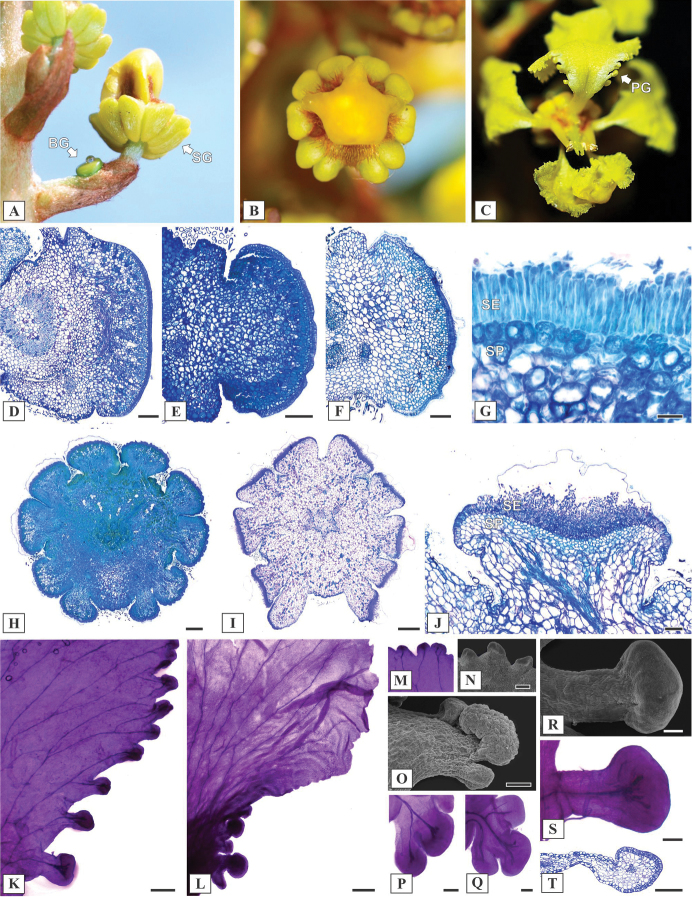
Reproductive morphoanatomy of *Mcvaughia* species. **A** inflorescence during development, showing a bracteole gland (BG) and Sepal glands (SG) **B** ten sepal glands encircling the calyx **C** Petal glands (PG) along the margin of posterior petal **D–F** transverse section of bracteole glands in *M.sergipana*, *M.bahiana* and *M.piauhiensis*, respectively **G** anatomical arrangement of bracteole gland, with a palisade-like secretory epidermis (SE) and secretory parenchyma (SP) **H–I** transverse section of floral bud and anthesis flower in *Mcvaughiabahiana* and *M.sergipana*; calyx gland pair displaced at the anterior sepal **J** calyx gland structure, showing a secretory epidermis (SE) and vascularized secretory parenchyma (SP) **K–L** petal glands on the margin of petals in *M.sergipana* and *M.bahiana* respectively **M–N** detail of the petal glands at the apex of the petal limb in *M.sergipana*, cleared and in SEM image **O–Q** petal glands positioned at the base, *M.bahiana* on SEM image, *M.bahiana* and *M.piauhiensis* cleared **R–T** conspicuous and stalked petal glands at the base of *M.sergipana*, in SEM image, cleared and longitudinal section. Scale bars: 200 μm (**D**), 150 μm (**E–F**), 50 μm (**G**), 500 μm (**H–I**), 100 μm (**J, P–S**), 300 μm (**L–M**), 200 μm (**N, T**).

Observations during field trips revealed that sepal and petal glands are yellow in *M.sergipana* and *M.bahiana* (Fig. 4A–B). The bracteole gland is initially green (Fig. 4A) turning yellow during anthesis. The secretory surface of the bracteole gland may vary from flattened (Fig. 4D) in *M.sergipana* to convex in the other two *Mcvaughia* species (Fig. 4E–F). The bracteole glands show an anatomical arrangement similar to that of the leaf glands, with palisade-like secretory epidermis and a vascularized secretory parenchyma (Fig. 4G). The calyces are particularly zygomorphic, due to the lateral displacement of glands in the anterior sepal (Fig. 4H–I). The bracteole glands are subsessile (Fig. 4D–F), while the sepal glands are short-stalked (Fig. 4J). Petal glands were recorded throughout the limb margin of the posterior petal in *M.sergipana* (Fig. 4K) and distributed only at the base in *M.bahiana* (Fig. 4L) and *M.piauhiensis*. In *Mcvaughiasergipana* the petal glands at the apex region are sessile (Fig. 4M–N). The basal petal glands in *M.bahiana* (Fig. 4O–P) and *M.piauhiensis* are sessile to slightly subsessile (Fig. 4Q), while in *M.sergipana* are stalked (Fig. 4R–S). The petal glands show the same anatomical composition as the leaf, bracteole, and sepal glands (Fig. 4T).

### Key to the species of *Mcvaughia*

**Table:** 

1	Leaf blades sericeous, margins revolute, several conspicuous and sessile glands near the midvein, straight outline of the wall in anticlinal epidermal cells, mesophyll with more than 4 layers of spongy parenchyma; cincinni 1–2-flowered; margins of posterior petal entirely glandular, staminode opposite the posterior petal with stout filament; restinga and coastal dunes	*** Mcvaughia sergipana ***
–	Leaf blades tomentose to lanate-velutinous, margins plain, conspicuous and stalked glands only near base and apex, sinuous outline of the wall in anticlinal epidermal cells, mesophyll with fewer than 4 layers of spongy parenchyma; cincinni (2–)3–7-flowered; margins of posterior petal glandular at base only, staminode opposite the posterior petal with slender filament; inland caatinga	2
2	Shrubs 1–3 m tall; leaf blades tomentose; flowers 1–1.2 cm diam., staminode opposite the posterior petal shorter than fertile stamens, apex of styles truncate; state of Bahia	*** Mcvaughia bahiana ***
–	Subshrubs ca. 50 cm tall; leaf blades lanate-velutinous; flowers 1.5–2 cm diam., staminode opposite the posterior petal as long as fertile stamens, apex of styles uncinate; state of Piauí	*** Mcvaughia piauhiensis ***

### 1. Mcvaughia bahiana

Taxon classificationPlantaeMalpighialesMalpighiaceae

W.R.Anderson, Taxon 28: 157. 1979

Figs 5 , 6 , 11

#### Type.

**BRAZIL. Bahia**: Conceição do Coité, road from Coité, 12 km to Santaluz, fl. Fr., 6 Mar 1976, W.R. Anderson 11740 (holotype: MBM barcode MBM59835!; isotypes: F barcode F0062743F!, G barcode G00352842!, K barcode K000426948!, MICH barcode MICH1102251!, NY barcode NY00067680!, P barcode P02429273!, RB barcode RB00540751!, SP barcode SP000249!, U barcode U0003826!, UB barcode UB1950!, US barcode US00108758!).

**Figure 5.**
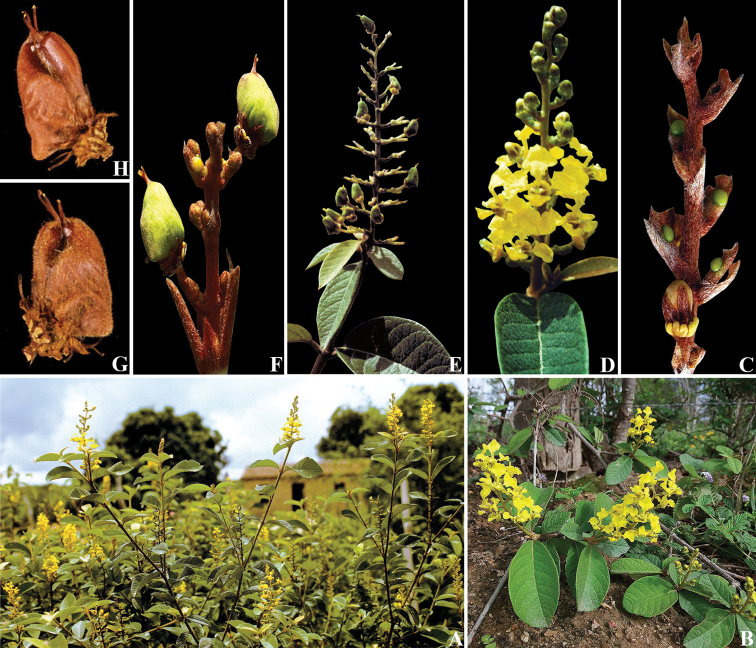
*Mcvaughiabahiana*. **A** shrub habit **B** subshrub habit **C** inflorescence showing glandular bracts **D** inflorescence showing flowers in anthesis **E** inflorescence showing immature and mature fruits **F** detail of mature fruits **G** drupe indumentum **H** glabrescent drupe. **A, D–H** by W.R. Anderson **B–C** by I.R. Guesdon.

**Figure 6.**
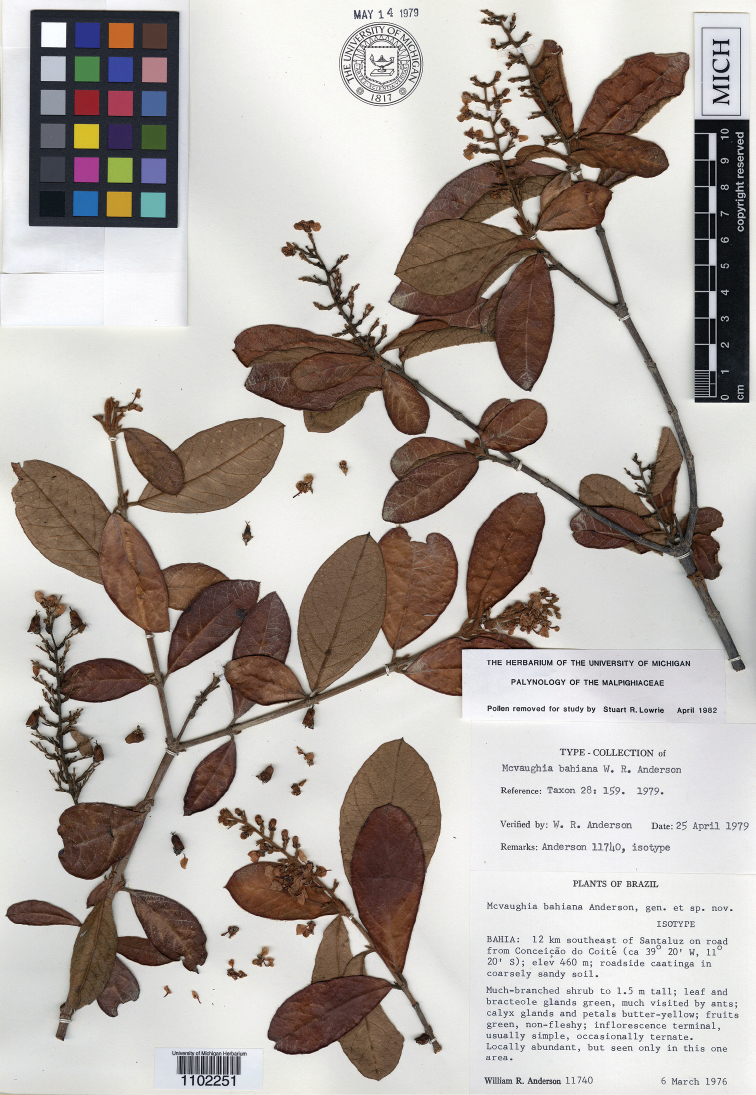
Photograph of the isotype of *Mcvaughiabahiana*.

#### Description.

*Shrubs* 1–3 m tall. *Branches* densely sericeous, glabrescent at age. *Stipules* 2.5–5 mm long, adaxially glabrous, abaxially sericeous. *Leaves* opposite; leaf blades 4.5–8.8 × 2–5 cm, chartaceous to subcoriaceous, elliptic to obovate, base cuneate to rotund, margins plain, entire, apex acute to apiculate, adaxial side initially tomentose, glabrous at age, abaxial side densely tomentose, a pair of conspicuous glands at base abaxially, on each side of the midrib, a few inconspicuous glands scattered over the blade, with 1–3 glands near the apex; petiole 0.3–0.7 cm long, canaliculate, densely tomentose, eglandular. *Thyrsi* of 2–7-flowered cincinni; rachis 3–10 cm long, smooth, densely tomentose, with brown hairs; lateral cincinni 12–24, subopposite; bracts 2–7 mm long, narrowly-triangular, appressed to the peduncle, eglandular, tomentose; peduncle 1.5–4.5 mm long, tomentose; bracteoles 1.5–2.5 mm long, narrowly triangular, opposite, appressed to the peduncle, tomentose, one of each pair bearing a conspicuous gland at base, 1–1.2 mm long. *Flowers* 1–1.2 cm diam. at anthesis, floral buds 3–3.2 mm long, pedicel 0.5–1.7 mm long, tomentose. *Sepals* 2–3 × 1.5–2.5 mm, straight, keeled, covering most of the androecium, apex acute, margin glabrous, adaxial side glabrous, abaxial side tomentose, glabrescent near the margin; 10-glandular, glands 1.5–2.5 × 0.7–0.8 mm, yellow, elliptic. *Petals* yellow, both sides glabrous, soon deciduous; anterior lateral petals orbicular, cucullate, nested inside one another, limb 3–3.3 × 4.5–4.7 mm, margin denticulate, 2-glandular at base, claws 1.2–1.5 × 0.2–0.25 mm, glabrous; posterior lateral petals obovate, spreading, limb 5.5–6.5 × 6–7 mm, margin erose, 2-glandular at base, claws 1.8–2 × 0.3–0.35 mm, glabrous; posterior petal obovate to orbicular, erect, limb 6–7 × 7–8 mm, margin erose, 3–5 pairs of rounded glands at the base of limb, proximal pair larger, claws 3–4 × 0.7–0.9 mm, both sides glabrous. *Stamens* free at base, filaments 2–3 × 0.2–0.4 mm, cylindrical, thicker at base; connective inconspicuous, glabrous; anthers 0.7–1 × 0.4–0.45 mm; staminodes opposite the posterior-lateral sepals covered by sepals, filaments ca. 1 mm long, long-triangular, anthers ca. 0.2 mm long, oblong, locules lacking; staminode opposite the posterior petal not covered by sepals, exserted, diverging from styles, filament 2–2.1 × 0.2–0.4 mm long, anther 0.25–0.3 mm long, oblong, locules reduced. *Ovary* 1–1.3 × 1–1.3 mm, ovoid, densely sericeous; styles 3, erect, ca. 2.5–2.7 × 0.5–0.6 mm, cylindrical, parallel, glabrous, apex truncate, anterior style slightly smaller than posterior ones; stigma lateral, circular. *Drupes* 7–8.5 × 4–5 mm, cylindrical, slightly twisted, apex with persistent styles, sparsely tomentose, with two chambers, proximal chamber containing the seed, distal chamber containing an oily substance; seed globose, smooth. *Embryo* not seen.

#### Specimens seen.

**BRAZIL. Bahia**: Itiúba, 20 km de Camaleão para Cansanção, 330 m, fl., 26 Feb 2000, A.M. Giulietti 1827 (CEN, FLOR, HUEFS, RB, UB); 20 Km East Camaleão, Rod. Itiúba/Cansanção, 21 Feb1974, fl., R.M. Harley 16465 (CEPEC, MICH, NY, P, RB). Monte Santo, Fazenda Bom Jesus, fl. fr., 11 Oct 2000, C.M.L. Aguiar 17, 18, 19, 27, 28, 30, 31 (HUEFS); fl. fr., 12 Jan 2006, M.L. Guedes 12148 (ALCB). Quijingue, Serra das Candeias, 5 Km W povoado Quixabá do Mandacaru, near Tucano, fl. fr., 15 May 2005, D. Cardoso 529 (HUEFS); fl. fr., 8 Jul 2006, D. Cardoso 1311 (HUEFS). Tucano, povoado Bizamum, 23 km from Tucano, fl. fr., 6 Jun 2004, D. Cardoso 57, 99 (HUEFS, SP); povoado Marizá, 13 km from Tucano, fl. fr., 6 Jan 2006, D. Cardoso 958 (HUEFS, RB); distrito de Caldas do Jorro, estrada entre Caldas do Jorro e rio Itapicurú, fl. fr., 1 Mar 1992, A.M. Carvalho 3863 (CEPEC, HUEFS, MBM, NY, SP); fl., 15 March 2008, G. Costa 341 (HST, HUEFS); povoado Bizamum, fl., 6 Feb 2004, L.P. Queiroz 9017 (HUEFS); povoado Bizamum, fl. fr., 22 Sep 2015, I.R. Guesdon 300, 301 (VIC).

#### Distribution, habitat, and phenology.

*Mcvaughiabahiana* is known only from sandy caatingas (seasonally dry forests) within northeastern state of Bahia, Brazil (Fig. 11). Flowering and fruiting throughout the year.

#### Conservation status.

*Mcvaughiabahiana* shows an extent of occurrence of 2,527 km^2^, and an area of occupancy of 16.000 km^2^ within the northeastern state of Bahia, Brazil. Its restricted distribution associated with an accelerated habitat degradation categorizes it as Endangered (EN). *Mcvaughiabahiana* is the only species in the genus not protected within the limits of a conservation unit.

#### Etymology.

The epithet refers to the distribution of *M.bahiana*, which is restricted to the state of Bahia, Brazil.

#### Anatomical notes.

Leaf glands are distributed throughout the leaf blade. Two basilaminar glands are typically positioned in pairs and visible to the naked eye (Fig. 3A). However, the anatomical study revealed a few additional glands distributed distally and difficult to see with the naked eye, two or three of them positioned subjacent to the apical leaf tooth. Anatomically, the basilaminar and laminar glands are short-stalked (Fig. 3D, J). The section of the bracteole and sepal glands revealed a subsessile anatomical structure (Fig. 4E). Malpighiaceous trichomes and their scars are frequent on the leaf, especially on the abaxial surface (Fig. 3O). On mature leaves, the indumentum along the middle and secondary veins and the apical leaf tooth is typically tomentose. The outline of the anticlinal walls is straight on the adaxial surface and sinuous on the abaxial (Fig. 3Q). Field observations revealed that the leaf glands are yellow, while the bracteole and sepal glands are green becoming yellow in blooming. The glands on the posterior petal are restricted to the proximal portion of the limb, where ca. 5 marginal glands were observed on each side of the petal limb (Fig. 4L, O–P).

### 2. Mcvaughia piauhiensis

Taxon classificationPlantaeMalpighialesMalpighiaceae

R.F.Almeida & Guesdon sp. nov.

urn:lsid:ipni.org:names:60478019-2

Figs 7 , 8 , 11

#### Diagnosis.

*Mcvaughiapiauhiensis* differs from *M.sergipana* Amorim & R.F.Almeida due to its leaf blades abaxially lanate-velutinous (versus sericeous), margins plain (versus revolute), conspicuous and stalked glands only near base and apex (versus throughout the leaf blade), cincinni 3–7-flowered (versus 2-flowered), margins of posterior petal glandular at base only (versus entirely glandular), and staminode opposite the posterior petal with slender filament (versus with stout filament). It also differs from *M.bahiana* W.R.Anderson due to its subshrub habit (versus shrub habit), leaf blades lanate-velutinous (versus tomentose), flowers 1.5–2 cm diam. (versus 1–1.2 cm diam.), staminode opposite the posterior petal as long as fertile stamens (versus shorter than fertile stamens), and the apex of styles uncinate (versus truncate).

**Figure 7.**
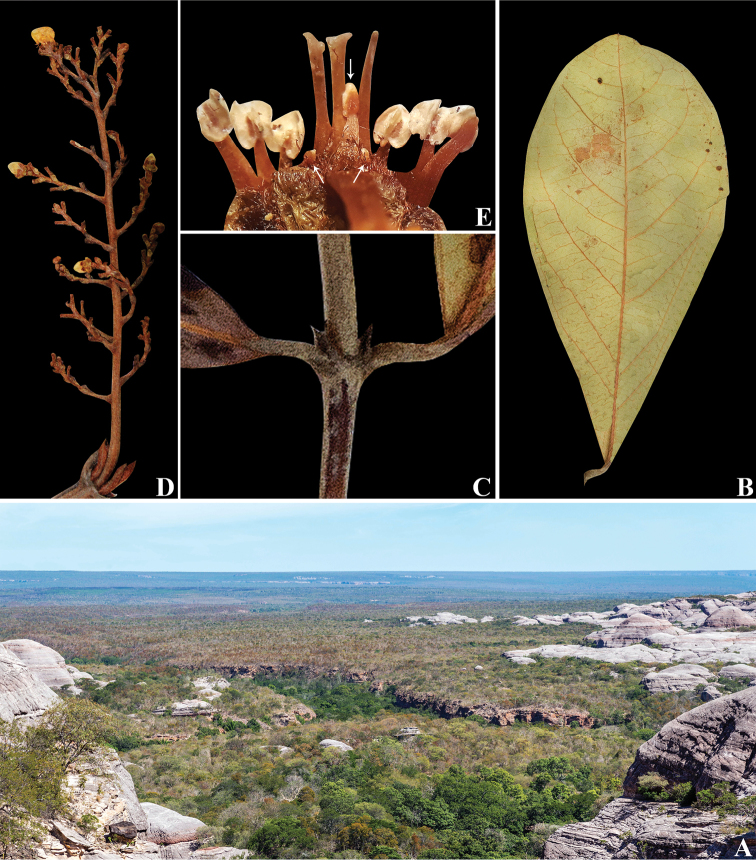
*Mcvaughiapiauhiensis*. **A** seasonally dry forests from Serra das Confusões, Piauí, Brazil **B** abaxial surface of a leaf **C** detail of epipetiolar stipules **D** inflorescence **E** rehydrated flower showing the stamens (white arrows= reduced stamens) and styles. **A** by S.E. Martins **B–E** by R.F. Almeida.

**Figure 8.**
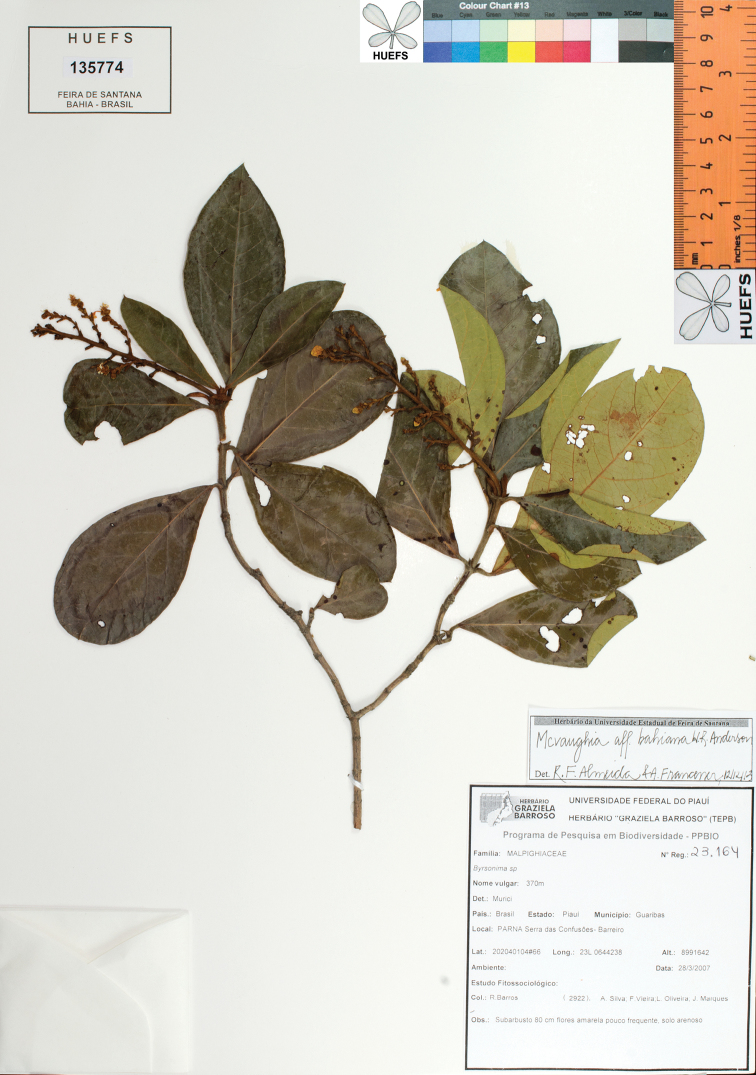
Photograph of the holotype of *Mcvaughiapiauhiensis*.

#### Type.

**BRAZIL. Piauí**: Guaribas, Parque Nacional da Serra das Confusões, Barreiro, fl., 28 Mar 2007, R. Barros 2922 (holotype: HUEFS barcode HUEFS135774!; isotype: CEPEC!, TEPB!).

#### Description.

*Subshrubs* ca. 50 cm tall. *Branches* densely lanate-velutinous, glabrescent at age. *Stipules* 4–4.5 mm long, adaxially glabrous, abaxially densely lanate-velutinous. *Leaves* opposite; leaf blades 6–11 × 3–6 cm, chartaceous to subcoriaceous, elliptic to obovate, base cuneate, margins plain, entire, apex acute to apiculate, adaxial side light green *in sicco*, initially lanate-velutinous to glabrescent, abaxial side dark green *in sicco*, initially lanate-velutinous to glabrescent, except from midvein at base, a pair of conspicuous glands at base abaxially, on each side of the midrib, a few inconspicuous glands scattered on the blade, with 2 conspicuous glands near apex; petiole 0.8–1 cm long, canaliculate, densely lanate-velutinous, eglandular. *Thyrsi* of 5–8-flowered cincinni; rachis 6.5–7 cm long, smooth, densely tomentose-velutinous, with rusty hairs; lateral cincinni 14–15, alternate; bracts 1.5–2.5 mm long, triangular, appressed to the peduncle, eglandular, tomentose-velutinous; peduncle 3–4 mm long, tomentose-velutinous; bracteoles 1.5–2 mm long, triangular, subopposite, appressed to the peduncle, tomentose-velutinous, one of each pair bearing a conspicuous gland at base, 1.3–1.8 mm long. *Flowers* 1.5–2 cm diam. at anthesis, floral buds 3–3.5 mm long, pedicel 2–3 mm long, tomentose-velutinous. *Sepals* 2–2.5 × 1–1.3 cm, straight, keeled, covering most of the androecium, apex rounded, margin short ciliate, adaxial side glabrous, abaxial side tomentose, glabrescent near the margin; 10-glandular, glands 1–1.2 × 0.7–0.8 mm, yellow, elliptic. *Petals* yellow, soon deciduous; anterior lateral petals orbicular, cucullate, nested inside one another, limb 2.9–3.2 × 4–4.3 mm, margin erose, eglandular, claws 1–1.2 × 0.2–0.25 mm, glabrous; posterior lateral petals obovate, spreading, limb 4–5 × 4–4.5 mm, margin erose, eglandular, claws 1.5–2 × 0.3–0.35 mm, glabrous; posterior petal obovate to orbicular, erect, limb 5–5.5 × 5–5.5 mm, margin erose, 2–3 pairs of reniform glands at the base of limb, proximal pair larger, claws 2.5–3 × 0.6–0.8 mm, adaxially pubescent. *Stamens* free at base, filaments 2–3 × 0.2–0.4 mm, cylindrical, thicker at base; connective inconspicuous, glabrous; anthers 0.3–0.45 × 0.4–0.45 mm; staminodes opposite the posterior-lateral sepals covered by sepals, filaments ca. 1 mm long, long-triangular, anthers ca. 0.2 mm long, oblong, locules lacking; staminode opposite the posterior petal not covered by sepals, exserted, diverging from styles, filament 2–2.1 × 0.2–0.4 mm long, anther 0.25–0.3 mm long, oblong, locules reduced. *Ovary* ca. 1 × 1 mm, ovoid, densely tomentose; styles 3, erect, ca. 3 × 0.5 mm, cylindrical, parallel, tomentose at base, uncinate at apex, anterior style slightly smaller than posterior ones; stigma lateral, circular. *Drupes* (immature) 5–6 × 2–3 mm, cylindrical, slightly twisted, apex with persistent styles, rusty tomentose, with two chambers, proximal chamber containing the seed, distal chamber containing an oily substance; seed (immature) globose, smooth. *Embryo* not seen.

#### Distribution, habitat, and phenology.

*Mcvaughiapiauhiensis* is known only from sandy caatingas (seasonally dry forests) within Serra das Confusões National Park in state of Piauí, Brazil (Fig. 11). Flowering in March.

#### Conservation status.

*Mcvaughiapiauhiensis* is known only from a single population within the limits of the Serra das Confusões National Park in state of Piauí, Brazil. Until additional fieldwork can be done in seasonally dry forests from Piauí, this species is best categorized as data deficient (DD).

#### Etymology.

The epithet refers to the distribution of *M.piauhiensis*, which is restricted to the state of Piauí, Brazil.

#### Anatomical notes.

This new species resembles *M.bahiana* in several aspects. The distribution pattern of leaf glands is quite similar, with both showing only one pair of conspicuous glands at base and a few conspicuous glands scattered over the blade (Fig. 3B). The basilaminar and laminar glands correspond to short-stalked glands (Fig. 3E, J). The sinuous outline of the anticlinal epidermal cell walls recorded on the abaxial leaf surface in *M.bahiana* (Fig. 3Q) was also observed in *M.piauhiensis*. On both surfaces of *M.piauhiensis*, the malpighiaceous hairs are less frequent (Fig. 3M), when compared with *M.bahiana* and *M.sergipana*, but the indumentum is clearly lanuginose-velutinous in young leaves. The distribution pattern of glands on the posterior petal is the same observed in *M.bahiana*, as ca. 5 short-stalked glands on the proximal portion of the petal limb margin (Fig. 4Q). Although no recent field observations have been recorded, we believe that the color of the glands and the color of the flower are the same observed in *M.bahiana*.

### 3. Mcvaughia sergipana

Taxon classificationPlantaeMalpighialesMalpighiaceae

Amorim & R.F.Almeida, Systematic Botany 40(2): 534. 2015

Figs 9 , 10 , 11

#### Type.

**BRAZIL. Sergipe**: Pirambu, estrada para lagoa redonda, restinga sobre tabuleiro, 10°41'79"S, 36°50'90.2"W, 96 m, fl. fr., 7 Oct 2013, A.M.A. Amorim et al. 8393 (holotype: CEPEC barcode CEPEC142146!; isotype: ASE barcode ASE0035770!, HUEFS barcode HUEFS226853!, MBM!, NY barcode NY02859382!, MICH!, P barcode P01168074!, RB barcodes RB01190994!, RB01191408!, RB01191409!, SP barcode SP003291!).

#### Description.

*Shrubs* 1.5–2 m tall. *Branches* densely lanate-velutinous, glabrescent at age. *Stipules* 3–5 mm long, adaxially glabrous, abaxially sericeous. *Leaves* opposite; leaf blades 8.4–12 × 2.7–6.5 cm, chartaceous to subcoriaceous, elliptic to ovate to elliptic-lanceolate, base acute to cuneate, margins slightly revolute, entire, apex acute to slightly acuminate, adaxial side green *in sicco*, initially sericeous to glabrescent, abaxial side metallic green *in sicco*, densely sericeous to glabrescent, 1–4 pairs of conspicuous glands at base abaxially, on each side of the midrib, with many conspicuous glands scattered distally; petiole 0.3–1.5 cm long, canaliculate, densely sericeous to glabrous at age, eglandular. *Thyrsi* of 1–2-flowered cincinni; rachis 6.5–11.4 cm long, striated, densely sericeous, with brown hairs; lateral cincinni 15–30, opposite to subopposite; bracts 5–6.5 mm long, lanceolate, spreading, eglandular, sericeous; peduncle 4–5 mm long, sparsely sericeous; bracteoles 2.5–3 mm long, triangular, subopposite, spreading to the peduncle, sericeous, one of each pair bearing a conspicuous green gland at base, 1.3–1.8 mm long. *Flowers* 1.5–2 cm diam. at anthesis, floral buds 3–3.5 mm long, pedicel 2–3 mm long, tomentose-velutinous. *Sepals* 2–2.5 × 1–1.3 cm, straight, keeled, covering most of the androecium, apex rounded, margin short ciliate, adaxial side glabrous, abaxial side tomentose, glabrescent near the margin; 10-glandular, glands 1–1.2 × 0.7–0.8 mm, yellow, elliptic. *Petals* yellow, soon deciduous; anterior lateral petals orbicular, cucullate, nested inside one another, limb 2.9–3.2 × 4–4.3 mm, margin erose, eglandular, claws 1–1.2 × 0.2–0.25 mm, glabrous; posterior lateral petals obovate, spreading, limb 4–5 × 4–4.5 mm, margin erose, eglandular, claws 1.5–2 × 0.3–0.35 mm, glabrous; posterior petal obovate to orbicular, erect, limb 5–5.5 × 5–5.5 mm, margin glandular, 2–3 pairs of stalked reniform glands at the base of limb, proximal pair larger and with many sessile glands scattered distally at the margin, claws 2.5–3 × 0.6–0.8 mm, adaxially pubescent. *Stamens* free at base, filaments 2–3 × 0.2–0.4 mm, cylindrical, thicker at base; connective inconspicuous, glabrous; anthers 0.3–0.45 × 0.4–0.45 mm; staminodes opposite the posterior lateral sepals covered by sepals, filaments ca. 1 mm long, long-triangular, anthers ca. 0.2 mm long, oblong, locules lacking; staminode opposite the posterior petal not covered by sepals, exserted, diverging from styles, filament 2–2.1 × 0.2–0.4 mm long, anther 0.25–0.3 mm long, oblong, locules reduced. *Ovary* ca. 1 × 1 mm, ovoid, densely tomentose; styles 3, erect, ca. 3 × 0.5 mm, cylindrical, parallel, tomentose at base, uncinate at apex, anterior style slightly smaller than posterior ones; stigma lateral, circular. *Drupes* 5–6 × 2–3 mm, cylindrical, slightly twisted, apex with persistent styles, rusty tomentose, with two chambers, proximal chamber containing the seed, distal chamber containing an oily substance; seed globose, smooth. *Embryo* not seen.

**Figure 9.**
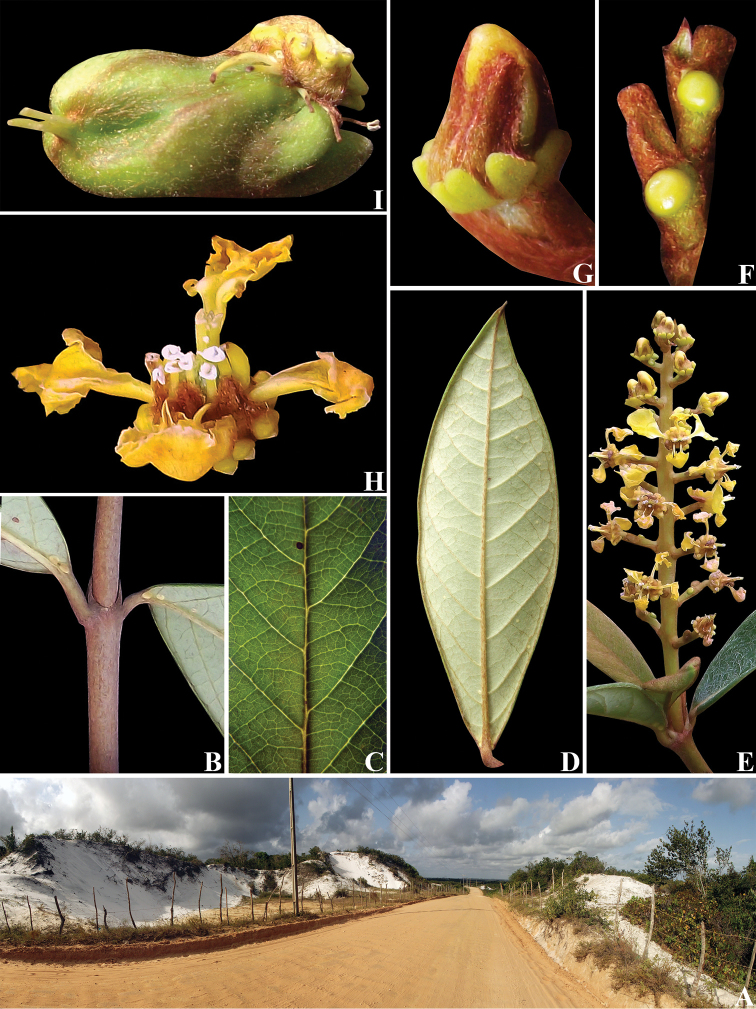
*Mcvaughiasergipana*. **A** sandy dune where *M.sergipana* occurs **B** detail of epipetiolar stipules **C** detail of leaf venation patterns **D** abaxial surface of a leaf **E** inflorescence showing buds and flowers **F** detail of glandular bracts **G** floral bud **H** flower in frontal view **I** fruit in side view. Photos by R.F. Almeida.

**Figure 10.**
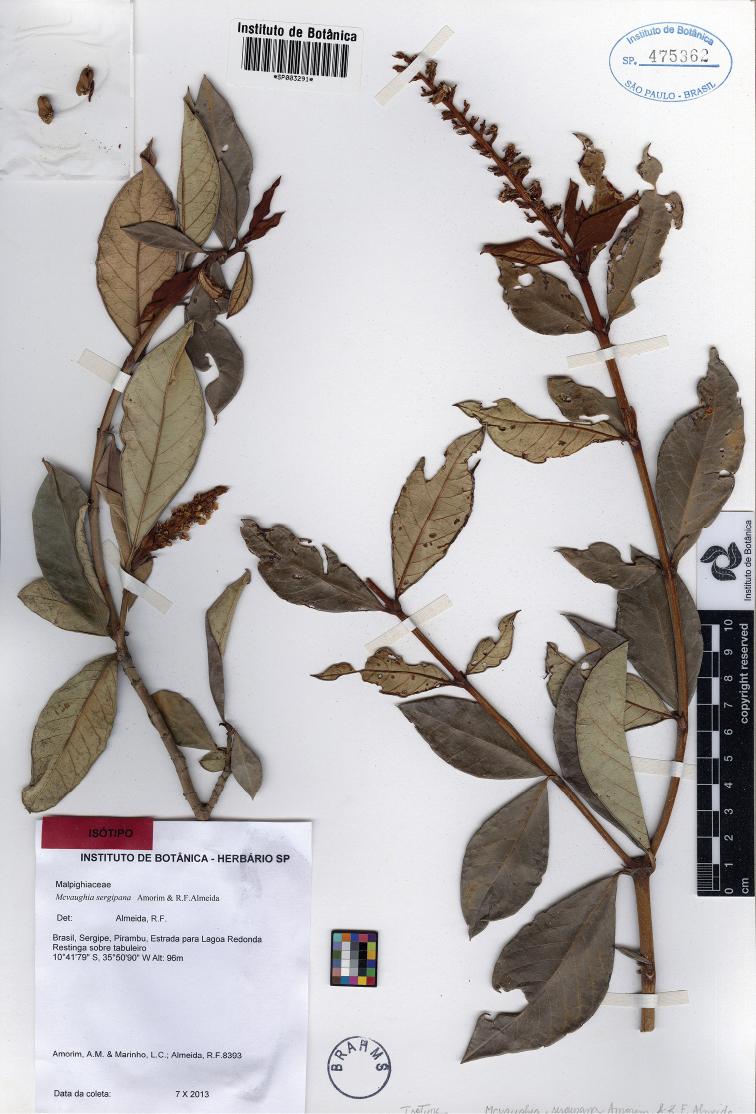
Photograph of the isotype of *Mcvaughiasergipana*.

#### Specimens seen.

**BRAZIL. Sergipe**: Japaratuba, povoado Sambaíba, fl. fr., 9 Sep 2013, B.C.A. Lima 37 (ASE); povoado Bonito, fl. fr., 24 Nov 2014, S.A. Damasceno 73 (ASE). Pirambú, estrada para Lagoa Redonda, fl. fr., 20 Dec 1978, M.R. Fonseca s.n. (ASE671); fl. fr., 17 May 2011, Santana 911 (ASE); fl. fr., 1 Nov 2011, E.A. Melo 13 (ASE); fl. fr., 9 May 2013, G.M.A. Matos 270 (ASE, CEPEC); fl. fr., 24 Sep 2015, I.R. Guesdon 305, 306 (VIC).

#### Distribution, habitat, and phenology.

**Figure 11.**
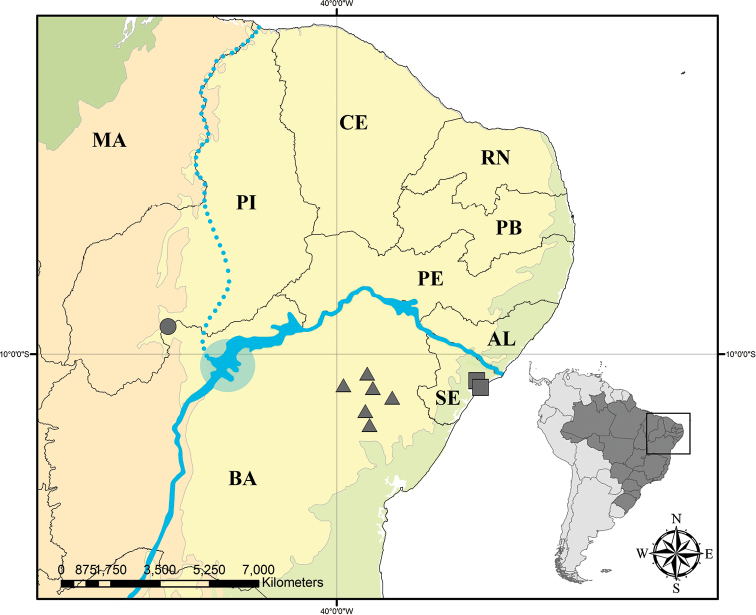
Distribution map of *Mcvaughia*: triangle – *M.bahiana*, circle – *M.piauhiensis*, and square – *M.sergipana*. Solid blue line in the center represents the São Francisco River today. Dotted blue line represents the past course of São Francisco River. Blue circle represents the São Francisco paleo lake. Light green – Atlantic Forest domain, dark green – Amazon Forest domain, orange – Cerrado domain, and yellow – Caatinga domain. AL – state of Alagoas, BA – state of Bahia, CE – state of Ceará, MA – state of Maranhão, PB – state of Paraíba, PE – state of Pernambuco, PI – state of Piauí, RN – state of Rio Grande do Norte, and SE – state of Sergipe.

*Mcvaughiasergipana* is known only from sandy restingas and coastal dunes within the Atlantic Forest Domain in the state of Sergipe, Brazil (Fig. 11). Flowering and fruiting from September to December.

#### Conservation status.

*Mcvaughiasergipana* shows an extent of occurrence of 49.735 km^2^ and an area of occupancy of 12.000 km^2^. Its restricted distribution and accelerated degradation of habitat categorizes it as Critically Endangered (CR). Even though the populations of *M.sergipana* are scattered within two municipalities in the state of Sergipe, Brazil, some of them are located within the limits of Santa Isabel Biological Reserve.

#### Etymology.

The epithet refers to the distribution of *M.sergipana*, which is restricted to the state of Sergipe, Brazil.

#### Anatomical notes.

This species has an unusual distribution pattern of leaf glands (Fig. 3C), in which all conspicuous glands are scattered throughout the leaf blade and visible to the naked eye. The basilaminar glands are generally positioned in pairs, varying from 2–8 glands. Several laminar glands are distally scattered throughout the blade, and one pair is positioned subjacent to the apical leaf tooth (Fig. 3G). The outline of the anticlinal epidermal cell walls is straight on both adaxial and abaxial surfaces (Fig. 3N, P). Leaf glands are yellow, and bracteole glands are green in secretory stage (Fig. 4A), turning yellow at blooming. The basilaminar and laminar leaf glands were anatomically identified as sessile (Fig. 3F, K), while the bracteole gland was recognized as subsessile (Fig. 4D) and the sepal gland as short-stalked (Fig. 4I). The bracteole gland shows a flattened secretory surface. Another important character that distinguishes *M.sergipana* is the distribution pattern of glands on the posterior petal. About five marginal petal glands are present on the proximal portion of the posterior petal in other *Mcvaughia* species, but only in *M.sergipana* do these glands extend the entire length of the petal margin (Fig. 4K, M–N). The proximal petal glands are stalked, in contrast to the small glands distributed distally that are sessile (Fig. 4R–T).

## Discussion on the anatomical characters

*Mcvaughia* can be characterized by very hard woods, narrow vessels in a radial arrangement, scanty axial parenchyma, heterocellular mixed rays, and large prismatic crystals in ray cells. The bark can be characterized by scattered fiber-sclereids, axial parenchyma in lines filled with druse crystals, intercalating with sieve tubes. *Mcvaughia* has various features similar to other Malpighiaceae, such as the narrow vessels in radial arrangement, simple perforation plates and simple sieve plates, minute vestured pits, parenchyma strands of 2–4 cells, heterocellular rays, and prismatic crystals in wood and druse crystals in the bark (Solereder 1908, Metcalfe and Chalk 1950, Amorim et al. 2017, Cabanillas et al. 2017, Pace et al. 2018). It is unique within the family because of its very scanty axial parenchyma, making it similar mainly to *Byrsonima*, which can also have a shrub habit (Solereder1908, Metcalfe and Chalk 1950). However, *Byrsonima* has shorter radial vessel chains, the rays are wider and heterocellular with body procumbent and square to upright marginal cells (Sonsin et al. 2014), as opposed to *Mcvaughia*, which has heterocellular mixed rays. *Byrsonima* also has abundant septate fibers, which are absent in *Mcvaughia*. From the two genera sister to *Mcvaughia*, namely *Burdachia* and *Glandonia*, only a few aspects are described in Solereder (1908), and Metcalfe and Chalk (1950) and these genera seem to diverge from *Mcvaughia* in having simple pits in the vessel-ray parenchyma interface, and *Burdachia* is described as having abundant paratracheal confluent parenchyma. The presence of heterocellular mixed rays is also unusual in this genus, since these types of rays are more common in lianas than in shrubs or trees (Amorim et al. 2017, Cabanillas et al. 2017, Pace et al. 2018) In the phloem, *Mcvaughia* is unique for the low abundance of sclerenchyma, which is generally quite abundant, at least in the nonconducting phloem of Malpighiaceae (Amorim et al. 2017, Cabanillas et al. 2017, Pace et al. 2018).

*Glandonia* species also show leaf glands varying from two to four basilaminar and a few to several laminar glands, which can be conspicuous or inconspicuous to the naked eye (Guesdon et al. 2018). Stalked glands have been recorded in the literature for *Banisteriopsis* (Araújo and Meira 2016; Nery et al. 2017), and *Stigmaphyllon* (Almeida and Mamede 2016); as well, sessile glands are known in *Diplopteryspubipetala* (Possobom et al. 2010), and sessile to immersed glands in *Amorimia* (Mello et al. in press.) and *Glandonia* species (Guesdon et al. 2018). Dorsiventral mesophyll with a single layer of palisade parenchyma and paracytic stomata are quite similar in all the three genera and commonly found in Malpighiaceae species, as reported by Araújo et al. (2010), Almeida et al. (2017), and Guesdon et al. (2018). The bracteole color observed in the field, could be used to distinguish the Mcvaughioid genera, being typically white in *Glandonia* (Guesdon et al. 2018), pink in *Burdachia* (Guesdon et al. unpubl. data) and green turning yellow in all *Mcvaughia* species. The secretory surface variation of the bracteoles glands in *Mcvaughia* provides diagnostic characters, as in species of *Glandonia* species (Guesdon et al. 2018). The short-stalked sepal glands recorded in species of *Mcvaughia* are also recorded in *Glandonia* (Guesdon and Meira unpubl. data), while subsessile sepal glands were reported in *Banisteriopsis* (Araújo and Meira 2016), and *Diplopteryspubipetala* (Possobom et al. 2015). The stalked and sessile petal glands found in *Mcvaughia* were also observed in *Burdachia* (Guesdon et al. unpubl. data), being only previously reported in *D.pubipetala* (Possobom et al. 2015). Leaf and bracteole glands have been described as true nectaries (Possobon et al. 2010; Araújo and Meira 2016, Nery et al. 2017; Guesdon et al. 2018). The sepal and petal glands have been identified as elaiophores on the sepals and as osmophores on the petals (Possobom et al. 2015; Araújo and Meira 2016; Guesdon and Meira unpubl. data). The anatomical similarity observed among all these glands support their homology, as suggested by molecular phylogenies (Castro et al. 2001; Davis and Anderson 2010).

The glands of the posterior petal provide promising floral characters for taxonomic studies in Malpighiaceae (Guesdon pers. com.). Previous studies in *Mcvaughia* (Anderson 1979; Amorim and Almeida 2015) have mentioned the presence of glands only at the proximal region of the posterior petal, and details about number, shape, and size are imprecise. Anatomic studies helped to elucidate the distribution of those structures, revealing not only the stalked glands on the proximal portion of the posterior petal, but also the sessile glands distally distributed exclusively in *Mcvaughiasergipana*. Additionally, the presence of glands on the lateral petals of *Mcvaughiabahiana* mentioned by Anderson (1979) was not confirmed in this study. However, this might be a result of intraspecific variation. The petal glands have also been taxonomically useful to distinguish species of *Glandonia* (Guesdon et al. 2018).

## Conclusions

The results presented in this study are the second step towards a complete taxonomic revision of the Mcvaughioid clade using several additional methods in biosystematics, initiated by Guesdon et al. (2018). Additionally, this is the first record of scanty sclerenchyma in the secondary phloem in Malpighiaceae, and of a shrub with heterocellular mixed rays, long radial rows of narrow vessels and scanty axial parenchyma in the wood. The macro and micromorphological data presented here are promising for future taxonomic and phylogenetic studies focusing on understanding the morphological evolution in the Mcvaughioid clade, and in Malpighiaceae, as well.

## Supplementary Material

XML Treatment for Mcvaughia

XML Treatment for Mcvaughia bahiana

XML Treatment for Mcvaughia piauhiensis

XML Treatment for Mcvaughia sergipana
